# The Differences in the Socio-Economic Levels and Blood Vessel Health States in Asian Countries

**Published:** 2019-06

**Authors:** So-Hyung KANG

**Affiliations:** Sports and Health Care Major, Mokwon University, Daejeon Metropolitan City, Korea

## Dear Editor-in-Chief

Recently, studies on the impact of economic growth on health outcomes have generated various conflicting results. In particular, these results get clear with regard to cardiovascular disease (1). Socioeconomic level affects the increase in cardiovascular disease prevalence (2). Nevertheless, the degree of impact of socioeconomic level and cardiovascular disease on study findings can be controlled through statistical or measurement methods. For instance, vascular stiffness measurements such as pulse wave velocity can independently predict the risk of cardiovascular disease (3).

Thus, we aimed to investigate the relationship between socioeconomic level, measured based on the gross national income (GNI), which represents the economic level index of a country, and vascular health, by measuring GNI and pulse wave delay time of 10 representative Asian countries.

The risk of cardiovascular disease was measured at K corporation in A city, GyeongGi province, Republic of Korea from June to September 2018. We included 1009 (males 505, females 504) individuals from 10 countries (China, India, Japan, Korea, Malaysia, Mongolia, Philippines, Singapore, Thailand, and Vietnam) who were currently living in Korea. The physical characteristics of the participants are detailed in Table 1. The participants provided informed consent for participation in the study.

**Table 1:** Characteristics of the participants

***Variables***		***Men(n=505)***	***Women(n=504)***
Age (yr)		43.24±6.41	41.86±5.08
Height (cm)		169.94±7.63	159.16±5.09
Weight (kg)		70.85±10.49	55.83±7.46
Body mass index (kg/m^2^)		24.27±2.38	22.04±2.82
Heart rate (beats/min)		69.98±8.94	70.60±13.19
Systolic blood pressure (mmHg)		117.79±10.34	115.00±4.12
Diastolic blood pressure (mmHg)		77.47±7.20	74.72±5.00
Pulse wave delay time	Right arm (msec)	195.10±14.93	207.72±33.17
	Left arm (msec)	194.98±15.40	201.07±18.95
	Right leg (msec)	293.85±30.10	298.46±29.81
	Left leg (msec)	292.04±29.09	297.99±30.33
Socio-economic levels	High (Japan, Korea, and Singapore)	152 (30.1)	153 (30.4)
	Middle (China, Malaysia, Mongolia, and Thailand)	204 (40.4)	201 (39.8)
	Low (India, Philippines, and Vietnam)	149 (29.5)	150 (29.8)

Results are expressed as mean±standard deviation or n (%)

Vascular compliance was measured for 3 minutes by attaching ECG ground sensors to the left and right forearms and covering pulse wave sensor with left and right index fingers and big toes after measuring the resting blood pressure during 10 minutes of rest. PWV-3.2 (KM–tec, Seoul, Korea) was used to measure Arterial-Pulse Wave Delay Time, for the analysis of vascular compliance. The Arterial-Pulse Wave Delay Time indicates the time that the QRS complex of cardiac ECG wave affects the arterial vascular pressure. Thus, an increase in Arterial-Pulse Wave Delay Time indicated an increase in the vascular compliance.

The economic level index was obtained from the 2018 GNI result value of World Bank (4). Gross national product **(**GNP) was measured as the sum of the gross domestic product (GDP**)** and the net abroad income, and GNI is sum of GDP, trade profit and loss according to change of terms of trade, and net abroad income. Namely, GDP is a product index and GNI is an income index.

Based on Arterial-Pulse Wave Delay Time result from the study and from the GNI of 10 Asian countries (4), Singapore, Japan, and Korea were classified as high economic level, Mongolia, Thailand, and Malaysia as middle economic level, and Vietnam, Philippines, and India as low economic level groups. The mean and standard deviation of the results were calculated using SPSS ver. 18.0 (SPSS, Chicago, IL, USA). One-way analysis of variance with Tukey test (post-hoc testing) was used to examine the difference of 3 groups by economic level and Arterial-pulse wave delay time, and statistical significance was set at *P<*0.05.

As shown in Table 2, there were no significant differences in the Arterial-Pulse Wave Delay Time according to the economic level in right and left arm and leg (*P*<0.05).

**Table 2:** Pulse wave delay time in Asian men and women by economic level

***Variable***	***Group 1***	***Group 2***	***Group 3***	***Overall F***	***Overall p***
Men	(n=152)	(n=204)	(n=149)		
Right arm (msec)	194.99±14.48	194.19±15.22 N/S	196.44±14.96 N/S	0.992	0.372 N/S
Left arm (msec)	193.77±14.76	194.30±15.06 N/S	197.17±16.35 N/S	2.182	0.114 N/S
Right leg (msec)	2290.78±27.43	293.21±29.26 N/S	297.86±33.41 N/S	2.196	0.115 N/S
Left leg (msec)	289.32±26.92	290.92±30.05 N/S	296.33±29.60 N/S	2.456	0.087 N/S
Women	(n=153)	(n=201)	(n=150)		
Right arm (msec)	206.13±32.73	207.91±33.86 N/S	209.08±32.82 N/S	0.303	0.738 N/S
Left arm (msec)	197.67±17.75	200.31±19.48 N/S	205.55±18.68 ^##^	6.984	0.001^**^
Right leg (msec)	295.28±34.93	299.92±27.27 N/S	299.75±27.23 N/S	1.251	0.287 N/S
Left leg (msec)	293.50±35.05	299.23±26.00 N/S	300.92±30.21 N/S	2.563	0.078 N/S

Note.

** *P<0.01*; one-way analysis of variance

N/S, not significant,

## *P*<0.01 compared with Group 1; Tukey’s post-hoc testing

Results are expressed as mean±standard deviation.

Group 1 = low economic level, Group 2 = middle economic level; Group 3 = high economic level

In females, except for left arm (*P*=0.001), there were no statistical significance in right arm and left right leg (*P*<0.05).

In conclusion, there was no specific difference of vascular health by economic level. Accordingly, it is assumed that vascular health, including economic level, is more related to other economy-related factors such as academic level, exercise practice rate, degree of knowledge of health, and dietary life.
